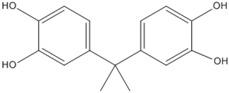# Photo-Oxidation of Bisphenol A in Aqueous Solutions at Near Neutral pH by a Fe(III)-Carboxylate Complex with Oxalacetic Acid as a Benign Molecule

**DOI:** 10.3390/molecules23061319

**Published:** 2018-05-31

**Authors:** Jing Xu, Chuxuan Zhao, Tianbei Wang, Shaojie Yang, Zizheng Liu

**Affiliations:** 1 State Key Laboratory of Water Resources and Hydropower Engineering Science, Wuhan University, Wuhan 430072, China; jingxu0506@whu.edu.cn; 2 Department of Environmental Science, School of Resource and Environmental Sciences, Wuhan University, Wuhan 430079, China; chuxuanzhao@whu.edu.cn (C.Z.); woshiyangshaojie@whu.edu.cn (S.Y.); 3 Engineering Research Center of Urban Disasters Prevention and Fire Rescue Technology of Hubei Province, School of Civil Engineering, Wuhan University, Wuhan 430072, China; 15071306559@163.com

**Keywords:** photo-oxidation, bisphenol A, Fe(III)-carboxylate system, oxalacetic acid, neutral pH

## Abstract

The photo-oxidation of organic pollutants as induced by ferric-carboxylate complexes was known to be a photo-Fenton-like process. The use of a carboxylate ligand with higher efficiency and lower toxicity at near neutral pH is of high interest to researchers. In this work, photo-oxidation of bisphenol A (BPA) induced by a ferric-oxalacetic acid complex in aqueous solutions was investigated under 395 nm LED lamps. The results showed that the rate of BPA degradation increased in the order pH 10.0 << 8.0 < 6.5 < 4.0 within the first 10 min. More than 90% of BPA was successfully oxidized with Fe(III)/oxalacetic acid with a ratio of 1:5 at pH 6.5, which was primarily attributed to the generated hydroxyl radical. Iron in the Fe(III)-oxalacetic acid system was reused by simple addition of oxalacetic acid to the reaction mixture. Compared to common carboxylate ligands (pyruvic acid, oxalic acid, and citric acid), oxalacetic acid is more efficient and environmentally friendly for the Fe(III)-carboxylate complex-based photo-Fenton-like process at near neutral pH.

## 1. Introduction

Ferric-carboxylate complex exhibits high photochemical activity via absorption of ultraviolet and visible light close to that of ambient sunlight (spectrum, 290–570 nm). Photolysis of the ferric-carboxylate complex results in an electron transfer from a ligand to the central metal (ligand metal charge transfer (LMCT)), producing ferrous ions and C-centered radicals. In the presence of oxygen, reactive oxygen species (ROS), O_2_^•^−/HO_2_^•^, H_2_O_2_, and HO^•^ are produced [1,2]. Similar to the Fenton reagent, Fe(III)-carboxylate complexes can produce active oxidant HO^•^ via photolysis, and have been widely used as advance oxidation processes (AOPs) for the degradation of organic pollutants [3,4,5,6]. Such ferric-carboxylate complexes-based photo-Fenton-like processes have two advantages making them environmentally friendly: (1) activation can be achieved by sunlight and (2) the reaction mixture does not require the addition of H_2_O_2_. However, in most cases, the pH is low, ranging from 3–5 [7], thus, the waste solution needs to be neutralized before emission. Consequently, it is of interest to find ligands that can work at near-neutral pH.

In the early 1990s, Zuo and Hoigné [1,2] reported the atmospheric photochemistry of ferric complexes with carboxylic acids (such as oxalic acid, pyruvic acid, and citric acid). From then on, these three ligands have been widely used for ferric-carboxylate complexes based on photo-Fenton-like processes. The work from Mailhot’s group on nitrilotriacetic acid (NTA) [8] and ethylenediamine-*N*,*N*′-disuccinic acid (EDDS) [9,10] provided ab new perspective in using alternative carboxylate ligands containing nitrogen. However, like ethylene diamine tetraacetic acid (EDTA), these nitrogen-containing carboxylates may pose a stress of eutrophication to receiving waters. Oxalacetic acid (OA), a keto acid like pyruvate, is an essential component of the Krebs cycle yielding citric acid and a metabolic intermediate from malic acid. OA is regarded as a non-toxic compound (LD_50_ > 5 g/kg, mouse) and no ecological toxicity of it has been reported, which implies that it is more environmentally friendly than oxalic acid. As far as we know, no photochemistry of the Fe(III)-oxalacetic acid (Fe(III)-OA) complex has been reported.

This work aims to confirm that oxalacetic acid can be used as a carboxylate ligand for the photo-Fenton-like process at near neutral pH. Both coordination chemistry and photochemistry of the Fe(III)-OA complex are presented herein. Bisphenol A (BPA) was selected as the model pollutant for degradation experiments because it is an endocrine disrupting substance and has been proven to be oxidized by various AOPs including Fenton and photo-Fenton oxidation [11,12,13].

## 2. Materials and Methods

### 2.1. Materials

OA (CAS: 328-42-7, 98%) and BPA (CAS: 80-05-7, 99%) were purchased from Sigma-Aldrich (Shanghai, China) and used as received. Ferric chloride (99%), hydrochloric acid, sodium hydroxide, 2-propanol, oxalic acid (Ox), pyruvic acid (Pyr), and citric acid (Cit) were obtained from Sinopharm Chemical Reagent Co., Ltd. (Shanghai, China). A stock solution of BPA in ultrapure water was prepared for photolysis experiments. All chemicals were of analytical reagent grade or higher purity and were used without further purification. Milli-Q water was used in all experiments.

### 2.2. Photolysis Experiments

Photolysis experiments were performed in a 200 mL open quartz beaker at ambient temperature under 395 nm LED lamps. The lamps were assembled as four plates around the beaker. Substrates at desired concentrations were prepared in a 200 mL flask and transferred into the beaker. The solution was stirred with a PTFE-coated magnetic stirrer. All solutions for the photolysis experiments were freshly prepared prior to irradiation. The pH value was quickly adjusted as needed using either an HCl or NaOH solution and measured by a pHS-3C meter. During the 1 h reaction time, samples were withdrawn at several intervals, and BPA concentrations were analyzed by high performance liquid chromatograph (HPLC).

To optimize reaction conditions, the initial pH, Fe(III), and OA concentrations were modified. To highlight the efficiency of OA, Ox, Pyr, and Cit were independently substituted for OA in three separate experiments, and the same procedure was followed as was used for reaction with OA. To determine the reactive species, isopropanol of different concentrations was added into the solutions to eliminate HO^•^. Oxidation experiments under sunlight were conducted with different solar irradiation intensities.

### 2.3. Determination of the Complexation Ratio and the Equilibrium Constant

UV-vis absorption spectra (200–600 nm) of the mixing solutions containing various concentrations of Fe(III) and OA were recorded with a 10-cm quartz cuvette, and absorbance at 390 nm was used to determine the complexation ratio and the equilibrium constant. The pH of the solutions was fixed in order to eliminate interference from any colloidal ferric hydroxide.

### 2.4. Analysis

The concentration of BPA was analyzed on a Shimadzu LC-10A HPLC system with an Agilent HC-C18 column (5 μm, 250 mm × 4.6 mm). The mobile phase was a solution containing water-methanol (30/70, *v*/*v*) at a flow rate of 1.0 mL min^−1^. The detector wavelength of bisphenol A was 278 nm. The LC-MS system used in the study was an Agilent LC/MSD SL ion trap mass spectrometer equipped with an ESI source in the negative ion mode. The mass spectral data were obtained in the positive ion mode between *m*/*z* 100–300. The settings were: capillary voltage, 3500 V; drying gas, 10 L/min; drying gas temperature, 350 °C; capillary exit, 150 V; skimmer, 40 V; octopole RF amplitude, 160 Vpp; ICC target, 100,000; trap drive, 60; maximum accumulation time, 100 ms.

## 3. Results and Discussion

### 3.1. Formation of Fe(III)-OA Complex

**Figure 1:**
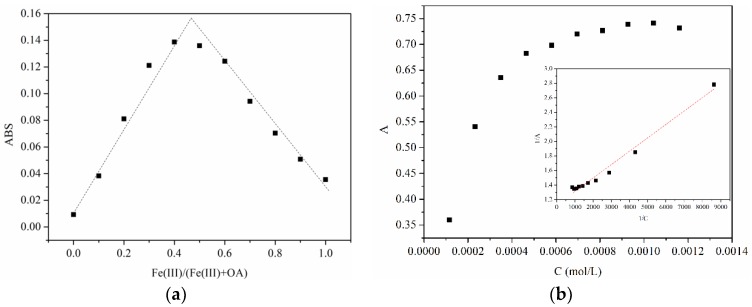
Changes in absorbance of (**a**) Fe(III)-oxalacetic acid (Fe(III)-OA) mixture solution with different Fe(III)/(Fe(III) + OA) ratios ([Fe(III)] = 0 − 116 μmol·L^−1^, [OA] = 116 − 0 μmol·L^−1^) and (**b**) Fe(III)-OA complex solutions with different OA concentrations ([Fe(III)] = 116 μmol·L^−1^, [OA] = 116 − 1160 μmol·L^−1^) at pH 2. Insert figure: linear fitting plot.

To confirm the formation of the Fe(III)-OA complex, absorbance of the Fe(III)-OA mixture solution at different Fe(III)/(Fe(III) + OA) ratios was measured in Figure 1a. The phenomenon followed an increasing but then decreasing pattern with a maximum value appearing at a Fe(III)/(Fe(III) + OA) ratio of around 0.5. This result reveals the existence of a 1:1 ligand-metal complex, which is similar to the previously reported Fe(III)-catechin complex [14].

Figure 1b shows the changes in absorbance of Fe(III)-OA complex with continuously increasing OA concentrations, which followed the Benesi-Hildebrand equation in Equation (1) [15], where *C_Fe_*^0^ and *C_OA_*^0^ are the initial concentration of Fe(III) and OA, *A* is the absorbance of solution, *K* is the equilibrium constant , and *ε* is molar absorption coefficient. As a result, the linearly fit equation was 1/A = 1.13 + 1.84 × 10^−4^ × 1/C, R^2^ = 0.981. The equilibrium constant for Fe(III) binding of the Fe(III)-OA complex was calculated as 6.12 × 10^3^ L·mol^−1^ via the linear fit. This equilibrium constant was much lower than that of a Fe(III)-Ox complex [1]. According to the calculation, the yield of the light quantum of Fe(II) in this system was 5.18 × 10^−2^.

(1) $$\frac{C_{Fe}^{0}}{A}=\frac{1}{C_{OA}^{0}}\times \frac{1}{\left(\varepsilon_{Fe-OA}-\varepsilon_{Fe}\right)K_{}}+\frac{1}{\left(\varepsilon_{Fe-OA}-\varepsilon_{Fe}\right)}$$

### 3.2. Photo-Degradation of BPA

**Figure 2:**
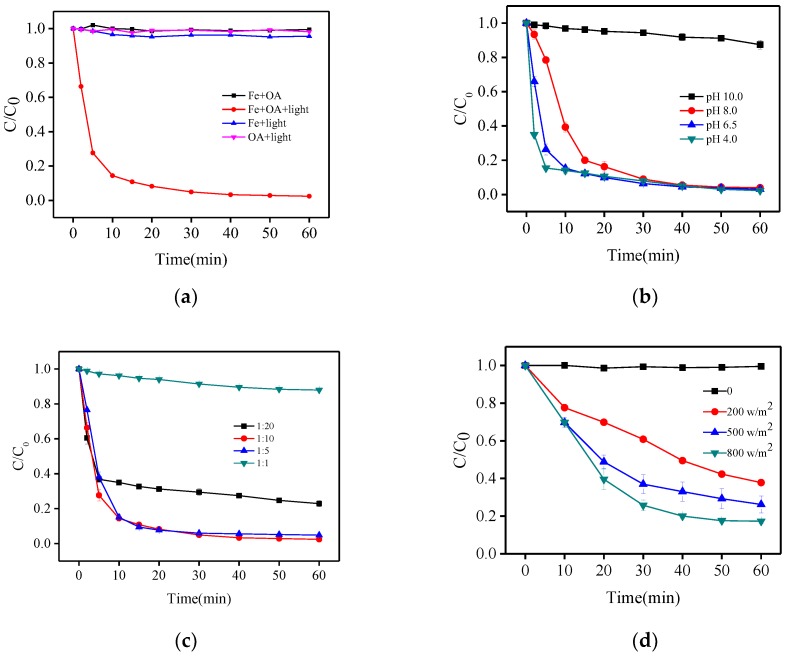
Photodegradation of bisphenol A (BPA), control experiments (**a**) and effect of initial pH (**b**), Fe(III)/OA ratio (**c**) and solar intensity (under sunlight) (**d**) on BPA degradation. [Fe (III)] = 116 μmol·L^−1^, [BPA] = 10 μmol·L^−1^, [OA] = 116 − 2320 μmol·L^−1^.

First, experiments with Fe(III) under light and Fe(III) in the dark under the same conditions (116 μmol/L Fe(III) and 580 μmol/L oxalacetic acid at pH 6.5) were conducted. The results of these experiments (Figure 2a) show that photo-degradation of BPA in the solutions with only iron or only oxalacetic acid at near-neutral pH were negligible. When Fe(III) and oxalacetic acid were used simultaneously without irradiation for 60 min, BPA degradation was negligible, indicating that the Fe(III)-OA complex could not be oxidized or adsorb BPA without light. Degradation of BPA in the Fe(III)-OA system was faster; ~97.6% BPA was removed after 60 min. Therefore, irradiation markedly enhanced BPA degradation. This enhancement may be attributed to the photoreactivity of the Fe(III)–OA complex.

To understand the performance of BPA degradation by a photo-Fenton-like process based on the Fe(III)-OA complex, experiments at different initial pH values, Fe(III)/OA ratios, and solar irradiation intensities (under sunlight) were carried out.

As shown in Figure 2b, the rate of BPA degradation increased in the order pH 10.0 << 8.0 < 6.5 < 4.0 within the first 10 min at a Fe(III)/OA ratio of 1:5, which is similar to the trend for atrazine degradation by an irradiated Fe(III)/Ox system [16]. It is noteworthy that the overall oxidation efficiency at 30 min was very approximate (91.0–92.1%) at pH ranging from 4.0–8.0. The results can be attributed to the following reasons: (i) the complex of iron and OA leads to greater stabilization of iron and exhibited effective photochemical activity at near neutral pH; (ii) Fenton-like reaction, i.e., the Fe(III)-carboxylate/Fe2+-H_2_O_2_ system always showed higher efficiency at acidic or near neutral pH, leading to a faster production of HO^•^ [17,18,19]; (iii) the formation of ferric hydroxide precipitate was enhanced at high pH. Only 5.6% of BPA was removed after 30 min of reaction time at pH 10.0. In this situation, Fe(III) existed as colloidal species and the formation of the Fe(III)-OA complex was negligible. Considering the real wastewater/water circumstance, near neutral pH 6.5 was selected for additional oxidation experiments.

Figure 2c shows the BPA degradation with various Fe(III)/OA ratios at pH 6.5. When the OA concentration was increased from 116 to 2320 μmol·L^−1^, the removal rate of BPA followed an increasing but then decreasing pattern. Up to 95.0–97.0% of BPA was successfully oxidized at a Fe(III)/OA ratio of 1:5 and 1:10, whereas BPA decreased by only 77.1% at a ratio of 1:20. This phenomenon may be explained as follows: on the one hand, increasing OA concentration could form more Fe(III)-OA complexes, resulting in the enhancement of generation of O_2_^•^−/HO_2_^•^, H_2_O_2_, and HO^•^ [20,21]; however, on the other hand, excess OA could rapidly eliminate HO^•^, leading to the significant decrease of BPA degradation [22,23]. Therefore, based on the economic cost and practicability of application, an optimal Fe(III)/OA ratio of 1:5 was selected for additional oxidation experiments.

The photo-oxidation of BPA under sunlight at pH 6.5 was also determined (Figure 2d). The removal efficiency of BPA after 60 min of irradiation remarkably increased from 62.2% to 82.7% with the solar intensity increasing from 200 to 800 W/cm^2^. This result demonstrated that the Fe(III)-OA complex utilized solar energy for the photo-Fenton-like process as well, which is in accordance with other Fe(III)-ligand complexes [24,25,26] and makes the process potentially economical and practical.

### 3.3. Role of Hydroxyl Radicals

**Figure 3:**
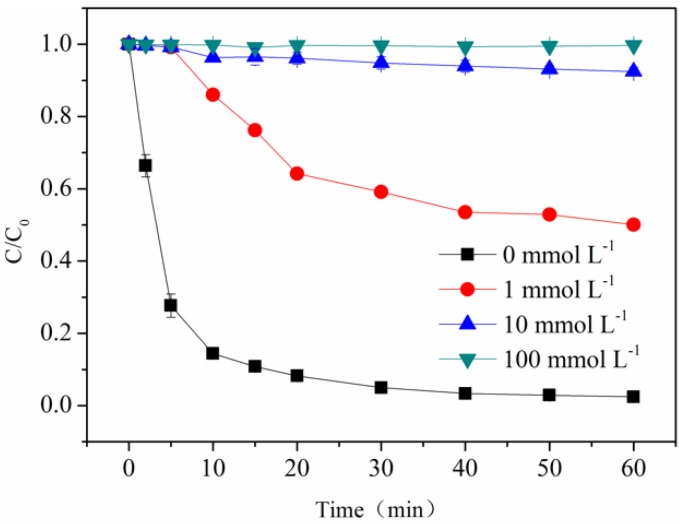
Effect of 2-propanol concentration on BPA photo-oxidation. [Fe(III)] = 116 μmol·L^−1^, [OA] = 580 μmol·L^−1^, [BPA] = 10 μmol·L^−1^, pH = 6.5.

In the photo-Fe(III)-ligand process, reactive radicals including O_2_^•−^/HO_2_^•^ and HO^•^ were always produced through an electron transfer from ligand to the central metal (LMCT) in the presence of oxygen [27,28]. For the purpose of identifying HO^•^ in the photo-Fe(III)-OA process, 2-propanol at various concentrations was introduced, because 2-propanol is an effective scavenger for HO^•^ (*k*_2-propanol,HO_^•^ =1.9 × 10^9^ M^−1^ s^−1^) [29]. Figure 3 shows that when 2-propanol concentration increased from 0 to 100 mmol·L^−1^ at pH 6.5, the removal efficiency of BPA was substantially reduced by 99.8%, indicating a marked inhibition effect of 2-propanol. The result implied that HO^•^ was produced in the photo-Fe(III)-OA process and subsequently contributed to BPA degradation. Moreover, owing to the fact that only 90.2% of BPA was degraded with an addition of 10 mmol·L^−1^ 2-propanol, HO^•^ was proposed to play a vital role for BPA photo-oxidation.

### 3.4. Reuse of Fe(III)/Fe(II) in the Solution

In the Fe(III)-OA system, the stability and solubility of Fe(III)/Fe(II) in aqueous solution at pH 6.5 was increased by addition of oxalacetic acid to form stable complexes. Iron catalyzes the reaction in the solution between the +2 and +3 oxidation states. However, overuse of iron will give rise to an increase of process cost and colored water. Therefore, it was necessary to reuse the Fe(III)/Fe(II) in the solution. In this regard, simple addition of oxalacetic acid to solutions provided a convenient approach.

**Figure 4:**
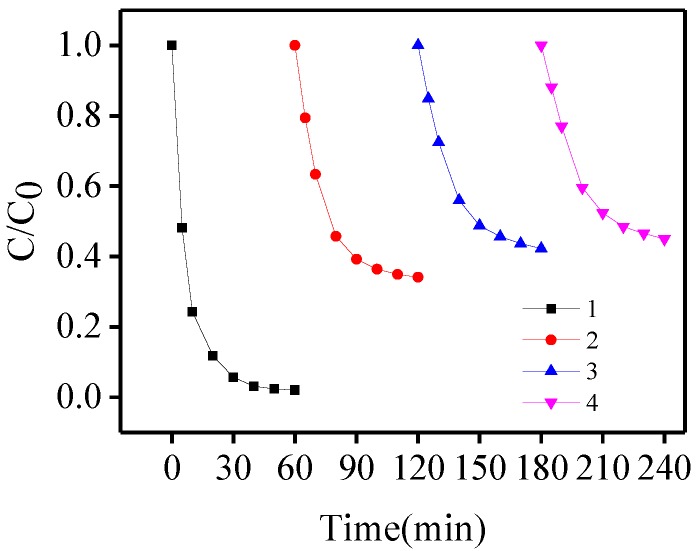
Photo-degredation of BPA after addition of oxalacetic acid (580 μM) to the Fe(III) solution (116 μM) in the four runs at pH 6.5.

In our study, four runs were conducted to investigate BPA photo-oxidation. Initially, the degradation was performed at pH 6.5 with 116 μmol/L Fe(III) and 580 μmol/L oxalacetic acid. After 60, 120, and 180 min, 580 μmol/L catechin was added to the solution. As shown in Figure 4, the degradation rate of BPA in the second run decreased by 65.9% compared with the first run, and slowly decreased in subsequent runs. This can be attributed to the accumulation of intermediates produced in the former run. Such intermediates may compete with ^•^OH for reaction with BPA in the solutions.

### 3.5. Photo-Degradation Products

Using LC-ESI-MS, we identified products of BPA photo-degradation in the Fe(III)-OA system to examine further the mechanism of BPA photo-degradation at pH 6.5. HPLC chromatograms and (–)-ESI-MS spectra of BPA and its photo-degradation products after 60 min of reaction are presented in Table 1. Two products with retention times of 7.8 and 5.7 min were identified. The *m*/*z* ratio of the photo-product at 7.8 min was 243.9 and that of it fragment ion was 227.7. It was possible that BPA could be added to a hydroxyl group. At 5.7 min, the *m*/*z* ratio of the photo-product was 260.5. It is possible that BPA can be added to two hydroxyl groups. One possibility was that an OH attack of side chains ending in their cleavage and oxidation led to the formation of this photo-product. The benzene series compounds were not detected in this experiment.

### 3.6. Comparison of Carboxylate Ligands

In order to further evaluate the general applicability of the Fe(III)-OA complex, an additional experiment was conducted by comparing OA with three different carboxylate ligands including oxalic acid (Ox), pyruvic acid (Pyr), and citric acid (Cit) in the photo-Fe(III)-ligand process.

**Figure 5:**
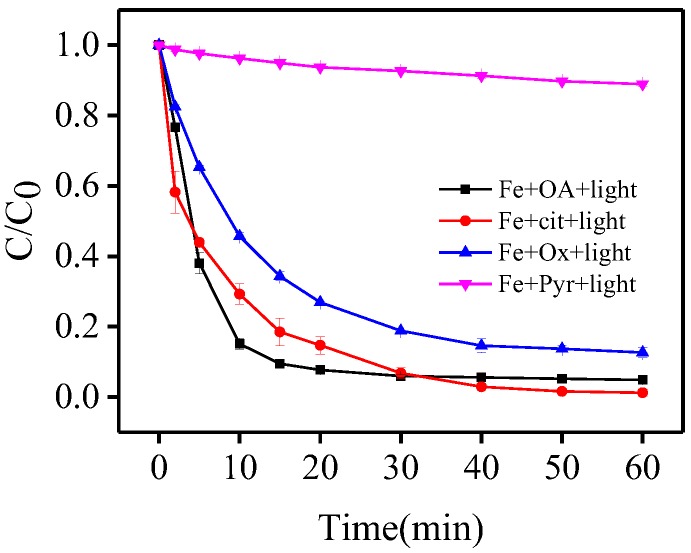
Comparison of different carboxylates of BPA photo-oxidation. [Fe(III)] = 116 μmol·L^−1^, [OA] = [Cit] = [Ox] = [Pyr] = 580 μmol·L^−1^, [BPA] = 10 μmol·L^−1^, pH= 6.5.

As shown in Figure 5, at a Fe(III)/ligand ratio of 1:5, the removal efficiencies of BPA reached 95.2%, 87.3%, 11.0%, and 98.8% for OA, Ox, Pyr, and Cit, respectively. The results established that the Fe(III)-OA complex had a high efficiency of BPA oxidation under the identical conditions, which may be related to the reaction mechanism in photo-Fe(III)-carboxylate system. In general, the ferric-carboxylate complex-based photo-Fenton-like processes always underwent a series of chain reactions [30,31], among which the reaction between Fe(II)-ligand and H_2_O_2_ was the critical process since the produced HO^•^ from such reaction was primarily responsible for the degradation of substrate. The different photochemical activity of the Fe(III)-carboxylate system could be explained as follows: (i) more hydroxyl groups in the structure of OA, Ox, and Cit; (ii) the asymmetric and unstable structure of OA and Cit may lead to the easier formation of the Fe(III)-ligand complex and subsequently generate more HO^•^. The negligible removal of BPA with Fe(III)-Pyr system was also attributed to ferric precipitation in the high pH range (pH ≥ 6.0) due to its weak coordination ability [32]. As a result, OA can be regarded as an efficient alternative ligand to induce a ferric-carboxylate complex-based photo-Fenton-like process.

## 4. Conclusions

Oxalacetic acid forms a 1:1 complex with ferric ions and has a characteristic absorption of light at wavelength ca. 390 nm. The photolysis of this complex induced production of HO^•^ that was responsible for oxidation of BPA in aqueous solutions at near neutral pH under irradiation of 395 nm LED light; the same process was also possible under sunlight. The main factors which influenced the process were the initial pH and Fe(III)/OA ratio. The Fe(III)-OA complex was superior to Fe(III)-Cit, Fe(III)-Ox, and Fe(III)-Pyr in photo-oxidation of BPA with the same Fe(III)/ligand ratio at pH 6.5. The attack of HO^•^ on BPA led to two degradation products. Oxalacetic acid acted as a benign ligand for the Fe(III)-carboxylate complex-based photo-Fenton-like process.

## Figures and Tables

**Table 1:** BPA and its major photolysis products in the Fe(III)-OA system by LC-MS analysis.

*m*/*z*	Retention Time (min)	Structure
228.6	11.2	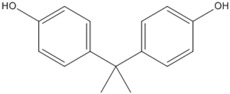
243.9	7.8	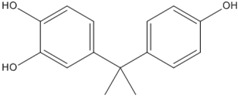
260.5	5.7